# Development of a Comorbidity-Based Nomogram to Predict Survival After Salvage Reirradiation of Locally Recurrent Nasopharyngeal Carcinoma in the Intensity-Modulated Radiotherapy Era

**DOI:** 10.3389/fonc.2020.625184

**Published:** 2021-01-20

**Authors:** Run-Da Huang, Zhuang Sun, Xiao-Hui Wang, Yun-Ming Tian, Ying-Lin Peng, Jing-Yun Wang, Wei-Wei Xiao, Chun-Yan Chen, Xiao-Wu Deng, Fei Han

**Affiliations:** ^1^Department of Radiation Oncology, Sun Yat-sen University Cancer Center, Guangzhou, China; ^2^State Key Laboratory of Oncology in South China, Guangzhou, China; ^3^Collaborative Innovation Center for Cancer Medicine, Guangzhou, China; ^4^Guangdong Key Laboratory of Nasopharyngeal Carcinoma Diagnosis and Therapy, Guangzhou, China; ^5^Department of Radiation Oncology, Hui Zhou Municipal Centre Hospital, Huizhou, China

**Keywords:** comorbidity, recurrent, nasopharyngeal carcinoma, reirradiation, prognostic nomogram

## Abstract

**Purpose:**

To assess the impact of comorbidity on treatment outcomes in patients with locally recurrent nasopharyngeal carcinoma (lrNPC) using intensity-modulated radiotherapy (IMRT) and to develop a nomogram that combines prognostic factors to predict clinical outcome and guide individual treatment.

**Methods:**

This was a retrospective analysis of patients with lrNPC who were reirradiated with IMRT between 2003 and 2014. Comorbidity was evaluated by Adult Comorbidity Evaluation-27 grading (ACE-27). The significant prognostic factors (P < 0.05) by multivariate analysis using the Cox regression model were adopted into the nomogram model. Harrell concordance index (C-index) calibration curves were applied to assess this model.

**Results:**

Between 2003 and 2014, 469 lrNPC patients treated in our institution were enrolled. Significant comorbidity (moderate or severe grade) was present in 17.1% of patients by ACE-27. Patients with no or mild comorbidity had a 5-year overall survival (OS) rate of 36.2 *versus* 20.0% among those with comorbidity of moderate or severe grade (P < 0.0001). The chemotherapy used was not significantly different in patients with lrNPC (P > 0.05). For the rT3–4 patients, the 5-year OS rate in the chemotherapy + radiation therapy (RT) group was 30.0 *versus* 16.7% for RT only (P = 0.005). The rT3–4 patients with no or mild comorbidity were associated with a higher 5-year OS rate in the chemotherapy + RT group than in the RT only group (32.1 and 17.1%, respectively; P=0.003). However, for the rT3–4 patients with a comorbidity (moderate or severe grade), the 5-year OS rate in the chemotherapy + RT group *vs.* RT alone was not significantly different (15.7 *vs.* 15.0%, respectively; p > 0.05). Eight independent prognostic factors identified from multivariable analysis were fitted into a nomogram, including comorbidity. The C-index of the nomogram was 0.715. The area under curves (AUCs) for the prediction of 1-, 3-, and 5-year overall survival were 0.770, 0.764, and 0.780, respectively.

**Conclusion:**

Comorbidity is among eight important prognostic factors for patients undergoing reirradiation. We developed a nomogram for lrNPC patients to predict the probability of death after reirradiation and guide individualized management.

## Introduction

Nasopharyngeal carcinoma (NPC) is a type of head and neck cancer. Globally, there were 129,079 new cases of NPC and 72,987 deaths reported worldwide in 2018 (1). Nevertheless, the geographical distribution is extremely unbalanced, and it is considered to be epidemic in East and Southeast Asia, and North Africa, and approximately >70% of new cases are in East and Southeast Asia (1, 2). Due to the concealed location and radiosensitivity of nasopharyngeal carcinoma, radiation therapy (RT) is the first-line treatment modality for primary NPC patients (3).

With the advancement of radiotherapy technology, the diagnostic imaging of nasopharyngeal carcinoma and the improvement of combined chemotherapy, the treatment effect and disease control rate have been improved significantly (4). Nevertheless, approximately 5–10% of patients experience locally recurrent nasopharyngeal carcinoma (lrNPC) after intensity-modulated radiotherapy (IMRT) (5–8). In patients with local recurrence, a repeat course of RT or nasopharyngectomy presents the only treatment options; however, to date, nasopharyngectomy is still challenging owing to its small space and previously irradiated anatomic space, which is often considered when feasible, or reirradiation for patients not eligible for surgery (9, 10). The emergence of IMRT has helped to overcome the technical limitations of conventional two- and three-dimensional RT and improved the therapeutic ratio of salvage RT; nevertheless, severe RT-related toxic reactions occur frequently and account for a large portion of mortality after IMRT treatment (up to 50%) (11, 12).

Hence, it has become evident that risk appropriately stratified *a priori* is warranted if individualized management is to be pursued for patients with lrNPC to avoid overtreatment. Sun et al. and Li et al. investigated robust prognostic models for risk stratification in lrNPC (13, 14). Nevertheless, Sun et al. only considered diabetes mellitus and hypertension among comorbidities, and Li et al. did not consider comorbidity as a prognostic factor. To address this issue, more accurate and comprehensive models are needed to identify individual risks by combining patient characteristics to assist with treatment recommendations in this patient subgroup with different risks. The ACE-27 is a modified Kaplan-Feinstein Index that assesses the severity of 27 different items related to cancer. A number of reports documenting the grading have shown reliability and validity in head and neck cancers as a cause of death, which have also been linked to predicting survival in head and neck cancers (15–18). In this study, we aimed to construct a nomogram that includes independent predictors. Moreover, we incorporated the ACE-27 into the nomogram to help clinicians predict patient survival outcomes and identify optimal candidates for local treatment with IMRT.

## Materials and Methods

### Patient Characteristics

A retrospective review of case records for patients with lrNPC who were reirradiated using a full-course IMRT technique was conducted at Sun Yat-sen University Cancer Center (SYSUCC) from January 2003 to December 2014. The inclusion criteria were as follows: a) pathology confirmed or evidence of local recurrence by at least one imaging study with a consistent clinical process, b) no evidence of distant metastasis, and c) using the IMRT technique for treatment. The exclusion criteria were a) pregnancy or lactation and b) secondary malignancy. The Clinical Research Ethics Committee of Sun Yat-sen University Cancer Center approved this study.

### Diagnosis and Comorbidity Assessment

Patients had undergone pretreatment evaluation comprising a complete medical history, nasopharynx and neck magnetic resonance imaging (MRI) or computed tomography (CT), chest X-ray or CT, abdominal CT or ultrasonography, CT whole-body bone scan single photon emission, or ^18^F-fluorodeoxyglucose (^18^F-FDG) PET/CT. ACE-27 was performed for comorbid disease severity at diagnosis, which includes the assessment of 27 elements from twelve different organ systems. The ACE-27 grades comorbidities into one of four scores: none 0), mild 1), moderate 2), or severe 3). The total score for each patient’s comorbidities is based on the highest-ranked single disease. When two or more moderate diseases occur in different organ systems, the total comorbidity score is considered severe (18–20).

### Clinical Treatment

A similar IMRT planning protocol was used as previously described (11, 21), and the total dosage for reirradiation therapy was 50–70 Gy at 1.80–2.50 Gy/fraction, five times a week on workdays, delivered by the IMRT technique. In this study, most patients received intravenous chemotherapy every 3 weeks. Treatment regimens included concomitant chemoradiotherapy plus induction chemotherapy (CCRT + IC), CCRT plus adjuvant chemotherapy (CCRT + AC), CCRT, RT, IC + RT, and RT + AC.

### Follow-Up and Endpoint

Follow-up was measured from the first day of therapy to the last follow-up or death. A range of assessments were carried out every 3 months for the first year, and then follow-up examinations were performed every 6 months thereafter until death. At each follow-up visit, routine assessments included head and neck physical examination, nasopharyngoscopy, nasopharynx and neck MRI with contrast, chest X-ray or CT, abdominal ultrasound or CT, whole-body bone scan, or (^18^F-FDG) PET/CT. The primary endpoint of our study was OS. OS was defined as the time from the first day of therapy to death from any cause or follow-up endpoint.

### Statistical Methods

Based on patient survival status, the optimal cut-off value of the continuous variables that generated the largest χ^2^ value in the Mantel-Cox test assessed by X-tile software (version 3.6.3; Yale University, New Haven, CT, USA), the lrNPC patients were stratified into subgroups (22). Life-table estimation was performed according to the Kaplan-Meier method. The log-rank test was used to examine the difference in survival between groups.

Multivariable regression analyses were performed using Cox proportional hazards modeling, which was used to estimate hazard ratios (HR) and 95% confidence intervals (CI), and formed the basis for the survival prediction model. Covariates in this study included comorbidity, age, sex, hemoglobin (HB), Karnofsky performance status (KPS), and prior RT-induced grade ≥ 3 toxicity variables (late RT-induced toxicities were noted and graded according to the Radiation Therapy Oncology Group radiation morbidity scoring scheme), DNA fragmentation index (DFI), recurrent gross tumor volume (GTV), rT stage, rN stage, treatment regimen, and re-RT equivalent dose in 2-Gy fractions [EQD2]. Those variables with a 2-tailed P < 0.05 in multivariable regression analyses were considered to create a nomogram based on their contribution to the accuracy of prediction. Statistical analyses were conducted using IBM SPSS Statistics software version 25, R version 3.6.1 (R Core Team, Vienna, Austria) and GraphPad Prism version 8.

## Results

### Patient Characteristics, Survival, and Toxicities

A total of 469 patients treated from January 2003 to December 2014 were retrospectively enrolled. The median age was 47 years old (range: 21–79), and 378 (80.6%) patients were male. A total of 19.6, 45.2, and 35.2% of the patients had stage rT1–2, rT3, and rT4, respectively, whereas 81.0 and 19.0% had stage rN0 and rN1–3, respectively, and 80 (17.1%) patients had prior RT-induced grade ≥ 3 toxicity. Comorbidity (moderate or severe grade) was present in 80 (17.1%) patients, and chemotherapy was delivered to 328 patients (69.9%). The median delivered EQD2 was 64 Gy [interquartile range (IQR): 61.10–67.10 Gy]. The other characteristics of the patients are detailed in **Table 1**.

**Table 1:** Clinical characteristics of 469 patients with locally recurrent nasopharyngeal carcinoma.

Characteristic	No. of patients (%) (n=469)
Sex	
Male	378 (80.6)
Female	91 (19.4)
Age (years) Media age	47
<60	399 (85.1)
≥60	70 (14.9)
Prior RT-induced grade ≥3 toxicity	
No	390 (83.2)
Yes	79 (16.8)
ACE-27 comorbidity grade	
0 or 1	389 (82.9)
2 or 3	80 (17.1)
KPS	
>70	453 (96.6)
≤70	16 (3.4)
HB (g/L)	
≥130	264 (56.3)
<130	205 (43.7)
Repeat IMRT EQD2,Gy	
Media Repeat IMRT EQD2,Gy	64
< 65	285 (60.8)
≥ 65	184 (39.2)
DFI (months)^a^	
≥60	104 (22.2)
<60	365 (77.8)
Recurrent T stage^b^	
rT1-2	92 (19.6)
rT3	212 (45.2)
rT4	165 (35.2)
Recurrent N stage^b^	
rN0	380 (81.0)
rN1-3	89 (19.0)
Volume of GTV-nx (cm3)	
<26	164 (35.0)
26-46	125 (26.7)
≥46	180 (38.4)
Target therapy^b^	
No	416 (88.7)
Yes	53 (11.3)
Chemotherapy	
No	141 (30.1)
Yes	328 (69.9)
IC alone	113 (24.1)
CCT alone	116 (24.7)
IC + CCT	93 (19.8)
AC alone	5 (1.1)
CCT + AC	1 (0.2)
IC + AC	0 (0)
IC + CCT + AC	0 (0)

RT, radiotherapy; ACE-27, Adult Comorbidity Evaluation-27; KPS, Karnofsky Performance Status; HB, hemoglobin; IMRT, intensity-modulated radiotherapy EQD2, equivalent dose in 2-Gy fractions; DFI, disease-free interval; GTV, gross tumor volume; AC, adjuvant chemotherapy; CCT, concurrent chemotherapy; IC, induction chemotherapy.

a DFI, disease-free interval was defined as the duration between the end of first RT and diagnosis of recurrence of > 6 months to exclude partial responders to the first course of RT.

b Target therapy includes cetuximab, nimotuzumab, and endostar.

All patients completed the IMRT treatment successfully, and the median follow-up was 36 months (range 3–193 months). The 3-year OS, locoregional relapse-free survival (LRRFS), and distant metastasis-free survival (DMFS) rates were 51.3, 70.0, and 90.0%, respectively; the 5-year OS, LRRFS, and DMFS rates were 33.4, 63.3, and 84.6%, respectively.

Treatment was not interrupted because of severe acute side effects in any patient. However, most patients experienced mild acute toxicities, including mucositis and xerostomia. Only 37 patients (7.9%) developed grade-3 acute toxicities. After reirradiation, 163 patients (34.8%) had one of the common late complications, including trismus, xerostomia, and hearing loss. A total of 141 patients (30.1%) had mucosal necrosis, 93 patients (19.8%) had temporal lobe necrosis, and 66 patients (14.1%) had cranial nerve palsy.

### The Impact of Comorbidity and Chemotherapy on Survival Outcomes

**Figure 1:**
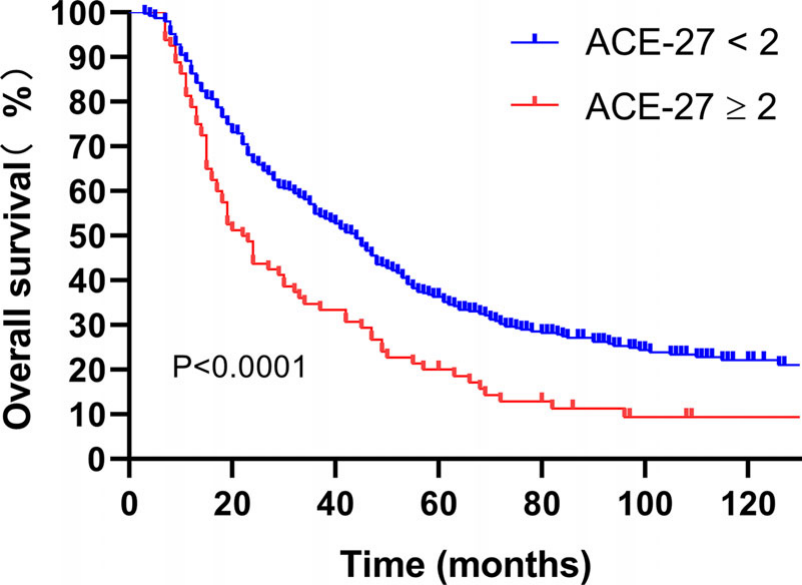
Kaplan-Meier analysis of overall survival is stratified by ACE-27. ACE-27, Adult Comorbidity Evaluation 27.

By the ACE-27 grading, of the 469 lrNPC patients, 188 (40.1%) had one or more comorbidities; 108 (23.0%) patients had ACE-27 scores of 1, 65 (13.9%) had scores of 2, and 15 (3.2%) had scores of 3. Patients with no or mild comorbidity had a 5-year OS rate of 36.2 *versus* 20.0% among those with comorbidity of moderate or severe grade (P<0.0001; **Figure 1**).

The prognosis of chemotherapy used was not significant in patients with lrNPC disease (P>0.05). Nevertheless, for the rT3–4 patients, the 5-year OS rate in the chemotherapy + RT group was 30.0 *versus* 16.7% for RT only (P=0.005). For rT3–4 patients with a comorbidity (ACE ≥ 2), the 5-year OS rate in the chemotherapy + RT group *vs.* for RT alone (15.7 *vs.* 15.0%, respectively; P>0.05) failed to confirm the positive association of chemotherapy. However, rT3–4 patients with an ACE-27 score of 0–1 had a higher 5-year OS rate in the chemotherapy + RT group than in the RT only group (32.1 and 17.1%, respectively; P=0.003). The results are presented in **Supplemental Figure S1**.

**Figure 2:**
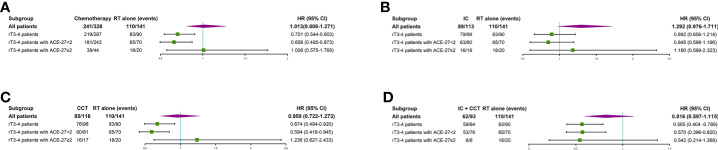
Subgroup analyses of overall survival for patients with radiotherapy only or chemotherapy plus radiotherapy **(A)**, overall survival for patients with radiotherapy only or induction chemotherapy plus radiotherapy **(B)**, overall survival for patients with radiotherapy only or concurrent chemotherapy plus radiotherapy **(C)**, and overall survival for patients with radiotherapy only or induction chemotherapy plus concurrent chemotherapy plus radiotherapy **(D)**. IC, induction chemotherapy; CCT, concurrent chemotherapy; CI, confidence interval; HR, hazard ratio.

A total of 141/469 (30.1%) lrNPC patients only received RT, 113/469 (24.1%) received IC + RT, 93/469 (19.8%) received IC + CCRT, 116/469 (24.7%) received CCRT, and only 6/469 (1.3%) received AC, including 5/469 (1.1%) who received RT + AC and 1/469 (0.2%) who received CCRT + AC. Due to the small number of AC patients, they were excluded following survival analysis. There was no significant difference between RT only and other treatment regimens (all P>0.05). However, for the rT3–4 patients, the 5-year OS rate in the RT group was 16.7 *vs.* 30.4% for CCRT (P=0.011), 38.6% for CCRT + IC (P = 0.001), and 20.7% for IC + RT (P>0.05). For rT3–4 patients with a comorbidity (ACE < 2), the 5-year OS rate in the RT group was 17.1 *vs.* 35.2% for CCRT (P=0.003), 38.8% for CCRT + IC (P=0.002), and 22.5% for IC + RT (P>0.05). However, for rT3–4 patients with a comorbidity (ACE ≥ 2), the 5-year OS rate in the RT group was 15.0 *vs.* 7.2% for CCRT, 37.5% for CCRT + IC, and 14.0% for IC + RT (all P>0.05) (**Supplemental Table S1** and **Supplemental Figure S2**). More details are shown in **Figure 2**.

### Univariate Analysis and Multivariate Analysis

Next, it was necessary to assess the prognostic significance of continuous variables and avoid any predetermined cutoff points. The X-tile analysis identified 1 optimal cut-off point, and the age, HB, EQD2, and DFI were 59 years, 128 g/L, 64.69 Gy, and 59 months, respectively. The analysis identified two optimal GTV cut-off points, 25.65 and 46 cc. For the use of minimum P statistics by Miller-Siegmund P-value correction, the best cut-off value is obtained (22). To optimize the cutoff value for its potential acceptance and clinical application, we rounded to the nearest integer in further analysis. Age (< 60 *versus* ≥ 60 years); HB (≤ 130 *versus* > 130 g/L); EQD2 (< 65 *versus* ≥ 65 Gy); and DFI (< 60 *versus* ≥ 60 months) were investigated. Similarly, the nearest integers of 26 and 46 cc were selected. In addition, based on clinical practice, a score of 70 was the cut-off value of KPS. Thus, we performed a univariable analysis on those variables that may be potential prognostic factors (**Table 2**). These variables were analyzed for association with OS by using Cox proportional hazards regression model hazard ratios (HRs), and the univariable P < 0.1 was included in the multivariable analysis. From the results of univariable analysis, age, prior RT-induced grade ≥ 3 toxicity, ACE-27, GTV, KPS, DFI, rT stage, and rN stage were significant survival predictors. On multivariate analysis, we found that age (P < 0.001), prior RT-induced grade ≥ 3 toxicity (P<0.001), KPS (P=0.003), ACE-27 (P=0.002), DFI (P=0.001), rT stage (P<0.001), rN stage (P=0.011), and GTV (P<0.001) remained independent prognostic factors.

**Table 2:** Univariable and multivariable analyses.

Characteristic	Univariable		Multivariable	
	Hazard ratio (95% CI)	P^a^ value	Hazard ratio (95% CI)	P^a^ value
Sex				
Male	reference			
Female	0.859 (0.657–1.124)	0.268		
Age (years)				
<60	Reference		Reference	
≥60	2.106 (1.606–2.763)	<0.001	2.189 (1.646-2.911)	<0.001
Prior RT-induced grade ≥3 toxicity				
No	Reference		Reference	
Yes	2.295 (1.765–2.986)	<0.001	1.923 (1.468–2.519)	<0.001
ACE-27 comorbidity grade				
0 or 1	Reference		Reference	
2 or 3	1.682 (1.293–2.188)	<0.001	1.553 (1.180–2.045)	0.002
KPS				
>70	Reference		Reference	
≤70	2.514 (1.521–4.155)	<0.001	2.213 (1.314–3.727)	0.003
HB (g/L)				
≥130	Reference			
<130	1.216 (0.985–1.501)	0.068		
Repeat IMRT EQD2, Gy				
<65	Reference			
≥65	1.186 (0.958–1.467)	0.117		
DFI (months)^b^				
≥60	Reference		Reference	
<60	1.348 (1.037–1.752)	0.026	1.585 (1.210–2.076)	0.001
Recurrent T stage				
rT1-2	Reference		Reference	
rT3	2.133 (1.543–2.948)	<0.001	1.692 (1.198–2.391)	0.003
rT4	3.238 (2.331–4.498)	<0.001	2.095 (1.429–3.070)	<0.001
Recurrent N stage				
rN0	Reference		Reference	
rN1-3	1.365 (1.058–1.761)	0.017	1.405 (1.082–1.824)	0.011
Volume of GTV-nx (cm^3^)				
<26	Reference		Reference	
26-46	1.947 (1.466–2.584)	<0.001	1.638 (1.210–2.216)	0.001
≥46	3.094 (2.384–4.015)	<0.001	2.295 (1.698–3.103)	<0.001
Target therapy^c^				
No	Reference			
Yes	1.321 (0.961–1.816)	0.087		
Chemotherapy				
No	Reference	0.908		
Yes	1.013 (0.808–1.271)			

RT, radiotherapy; ACE-27, Adult Comorbidity Evaluation-27; KPS, Karnofsky Performance Status; HB, hemoglobin; IMRT, intensity-modulated radiotherapy EQD2, equivalent dose in 2-Gy fractions; DFI, disease-free interval; GTV, gross tumor volume; CI, confidence interval.

a P values were calculated using Cox proportional hazards model.

b DFI, disease-free interval was defined from the date of completion of treatment to diagnosis of recurrence or final follow-up if sooner.

c Target therapy includes cetuximab, nimotuzumab and endostar.

The following variables were included in the Cox proportional hazards model with forword LR elimination: the age (≥ 60 vs. < 60); KPS (≤ 70 vs. > 70); HB (≥ 130 vs. < 130); DFI (≥ 60 vs. < 60), prior RT-induced grade ≥ 3 toxicity (yes or no); recurrent T stage (T1–2 vs. T3 vs. T4); recurrent N stage (N0 vs. N1–3); GTV (< 26 vs. 26–46 vs. ≥ 46).

### Establishment and Evaluation of a Nomogram Model for Overall Survival

**Figure 3:**
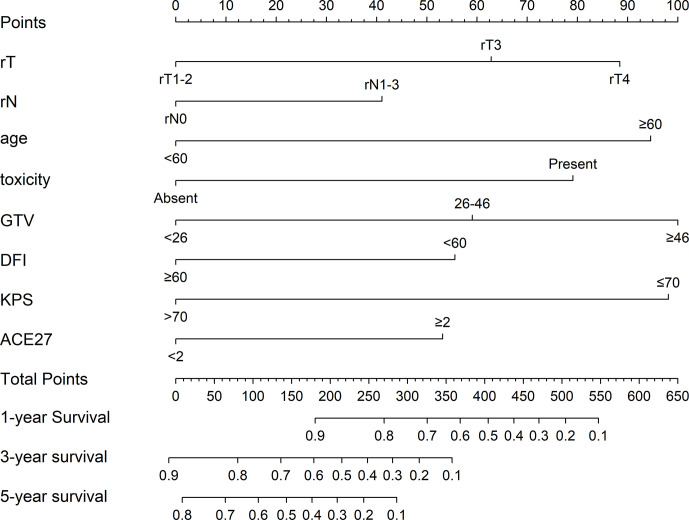
Prognostic nomogram for locally recurrent nasopharyngeal carcinoma (lrNPC) patients: a line was drawn straight down to predict the 1-, 3-, or 5-year overall survival. rT, recurrent T stage; rN, recurrent N stage; toxicity, prior RT-induced grade ≥ 3 toxicity; GTV, gross tumor volume; DFI, disease-free interval; KPS, Karnofsky Performance Status; ACE-27, Adult Comorbidity Evaluation-27.

**Figure 4:**
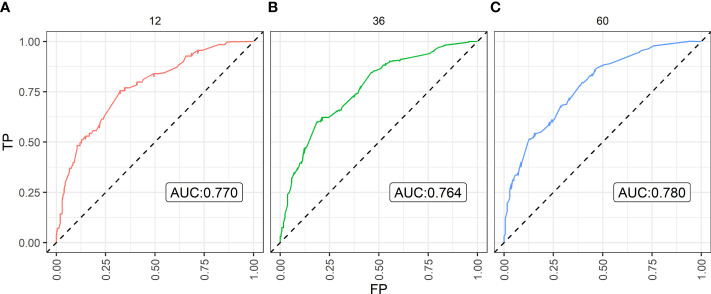
The Area Under Curve (AUC) of the prediction nomogram on **(A)** 1-year, **(B)** 3-year, and **(C)** 5-year overall survival. TP, true positive; FP, false positive.

Based on the eight independent prognostic factors, a nomogram was established for predicting the 1-, 3-, and 5-year OS for lrNPC (**Figure 3**). Each variable has a corresponding score according to the point scale, and we obtained the total score by calculating the score of each variable. Next, by mapping the total score on the probability scale, the OS probabilities could be estimated at the 1-, 3-, and 5-year time points (**Figure 4**)

By bootstrap correction, the Harrell C index was 0.715 in the nomogram. The area under curves (AUCs) for the prediction of 1-, 3-, and 5-year OS were 0.770, 0.764, and 0.780, respectively. The results exhibited satisfactory accuracy for predicting the 1-, 3-, and 5-year OS for lrNPC. The calibration curves showed that the nomogram predictions were well correlated with the actual observations for 1-, 3-, and 5-year OS (**Supplemental Figures S3–S5**).

## Discussion

In general, ACE-27 in patients with primary NPC has been reported, and it has been explained to be a major determinant of survival outcome in patients with NPC (16, 23, 24). To our knowledge, the value of ACE-27 in patients with lrNPC has not yet been analyzed, and this study is the first to describe ACE-27 in patients diagnosed with lrNPC and to assess the prognostic value of ACE-27 on the survival of patients with lrNPC. In this study, comorbidity was present in 40.1% of patients. The incidence of comorbidity in lrNPC patients is similar to that in primary NPC patients (17). However, patients with moderate and severe comorbidities were observed to have higher rates of comorbidity than primary NPC. The reason may be that the patients become susceptible to disease or the original comorbidity has been aggravated after the initial radiotherapy and chemotherapy. The patients with an ACE-27 score of 0–1 had a better survival than those patients with an ACE-27 score of 2–3. This result is similar to that previously reported in primary NPC (24). In addition, patients who had rT3–4 patients with ACE-27 scores of 0–1 who received chemotherapy had better survival than those with RT only. Nevertheless, there was no significant difference between RT only and RT + chemotherapy in patients who had rT3–4 patients with ACE-27 scores of 2–3. Our findings suggest that patients with ACE-27 scores of 2–3 cannot benefit from chemotherapy. The reason may be that those patients have a shorter median survival time, and the benefits of chemotherapy have not been observed; furthermore, comorbidity increases the risk of death from other diseases. It can explain the negative impact of rT3–4 with comorbidities (moderate or severe) on survival.

Although various chemotherapy regimens and targeted agents have been used, there is still a lack of effective clinical evidence for the use of chemotherapy in lrNPC patients (25–27). A meta-analysis did not demonstrate that the addition of chemotherapy had an impact on local failure-free survival (LFFS), DMFS, and OS (28). In our study, however, we found that rT3–4 patients who received CCRT + IC or CCRT had better survival than those who received RT only, but those who received IC + RT had no significant OS benefit compared to those who received RT alone. In particular, rT3–4 patients with a comorbidity (ACE < 2) have similar outcomes. The reason may be that concurrent chemotherapy can improve radiosensitivity, thereby improving local tumor control. Nevertheless, patients with larger GTVs often choose to have increased IC. In addition, IC related to increased associated late toxicities should also be considered. The results show that CCRT may be a preferential therapeutic regimen. It is still worth exploring whether IC can benefit lrNPC patients. Ng WT et al. reported that IC followed by CCRT and weekly cetuximab could achieve a better treatment outcome (3-year PFS and OS rates of 36 and 64%, respectively) in lrNPC (T3–T4, N0–N1, M0) patients than reported in previous studies (29). The sample size in this study is small. To obtain high-level evidence, a prospective, multicenter, phase III clinical trial should be implemented. In addition, some studies have reported that immunotherapy has acquired promising results in recurrent or metastatic nasopharyngeal carcinoma patients (30–32). Nevertheless, the studies included only a small number of lrNPC patients. The effect of immunotherapy deserves more exploration by a large-scale clinical trial of lrNPC.

Some recent studies have shown that the dose of reirradiation by IMRT is an essential prognostic factor in patients with lrNPC (11, 14, 33, 34). In a prognostic model proposed by Li et al. (14), the dose of reirradiation by IMRT (EQD2 ≥ 68 Gy) is a poor prognostic factor. It is combined with the other four factors to form a prognostic index (PI). Tian et al. demonstrated that decreasing the total dose and increasing the fraction size can achieve local control similar to that achieved with a higher dose after IMRT, and it can improve OS by reducing the incidence of severe late complications (33). However, there are small samples in this study. Another study by Ng WT et al. (34) reported that a reirradiation dose equivalent to 60 Gy (EQD2) appears to be the optimal dose for achieving the best survival outcome while balancing the probability of local control and fatal complications. Those studies indicated that 60 Gy may be an optimal dose for lrNPC patients, but these studies are based on the analysis of small samples, and the patients in the study by Ng WT reported came from multiple centers. There are great differences in treatment strategies and dose limitation standards. Moreover, in our retrospective study, EQD2 (< 65 *versus* ≥ 65 Gy) had no significant effect on survival; however, the incidence of mucosal necrosis in the higher EDQ2 group was higher than that in the lower EQD2 group (40.2 *versus* 23.5%; respectively, P<0.001) and temporal lobe necrosis (25.5 *versus* 16.1%; respectively, P=0.013). This means that the high reirradiation dose increases the occurrence of complications. We consider that, with dose escalation, the survival benefit is potentially offset by survival detriment because of increased late complications. A meta-analysis by Leong YH found that DFI ≥ 36 months can obtain a higher rate of LFFS, but there is no benefit in OS and DMFS (28). In our study, DFI was an important prognostic factor. The cut-off time was 60 months, and the longer DFI may allow the patient’s previously irradiated organs to recover better and reduce the degree of damage by radiotherapy.

In addition, age, rT stage, and GTV were the most important predictors of OS in previous reports (11, 14, 28). We can obtain similar results in this study. To distinguish the impact of GTV on prognosis, we obtained two cutoff values using X-tile software, and the results showed that the different GTV groups had significantly different survival prognoses. The cut-off value of hemoglobin in this study was obtained using the software X-tile and the value was 128 g/L. However, in order to facilitate the application of the nomogram in clinical practice, we selected 130 g/L as the cut-off value finally. There are great variations between the selected cut-off value of hemoglobin in the previous studies, and there is no uniform standard so far (35–37). Some reports showed that relatively low levels of hemoglobin is a poor prognostic factor for nasopharyngeal carcinoma (35, 36). However, in our study, we fail to get the similar results.

Moreover, the present study has several limitations that deserve further discussion. First, plasma Epstein-Barr virus (EBV) DNA was regarded as an adverse prognostic biomarker in primary NPC (38). In this study, we excluded EBV DNA from the nomogram. We considered the positive rate of pretreatment plasma EBV DNA in the detection of lrNPC to be relatively low, and the prognostic significance of EBV DNA, which lacks prospective data in lrNPC, was uncertain. In addition, the method of plasma EBV DNA measurement lacked standardization, and the EBV DNA polymerase chain reaction assay is susceptible due to changes in experimental conditions (39). Second, it is a single-center retrospective study in an endemic area, and some amount of selection bias is unavoidable. Finally, our proposed models were not validated in an external cohort, and the C-indices of the nomogram showed an imperfect discrimination ability, which indicated that more factors should be considered. Nonetheless, we acknowledge these limitations of our model. The focus of this study is that it is the first large-scale study to evaluate the importance of ACE-27 and its value in predicting survival for lrNPC.

## Conclusion

In summary, our study is a large-scale study of lrNPC treated with IMRT. In this study, eight prognostic factors are worth investigating before reirradiation. For patients with favorable risk factors, reirradiation should be strongly recommended because it may have better survival benefits. However, for patients with high-risk factors, palliative chemotherapy or immunotherapy should be considered.

## Data Availability Statement

The raw data supporting the conclusions of this article will be made available by the authors, without undue reservation.

## Ethics Statement

Written informed consent was obtained from the individual(s) for the publication of any potentially identifiable images or data included in this article.

## Author Contributions

FH, X-WD, and C-YC provided the study concepts. R-DH, ZS, and X-HW designed the study. R-DH, ZS, and X-HW acquired the data. Y-MT, Y-LP, J-YW, and W-WX conducted the quality control of the data and algorithms. R-DH, ZS, and X-HW analyzed and interpreted the data. R-DH, ZS, and X-HW conducted the statistical analysis. R-DH, ZS, and X-HW prepared the manuscript, R-DH, ZS, and X-HW edited the manuscript. FH, X-WD, and C-YC reviewed the manuscript. All authors contributed to the article and approved the submitted version.

## Funding

This work was supported by the National Natural Science Foundation of China (12005316), Cancer Precision Radiotherapy Spark Program of China International Medical Foundation (2019-N-11-20), and Medical Scientific Research Foundation of Guangdong Province, China (Granted No. A2016197).

## Conflict of Interest

The authors declare that the research was conducted in the absence of any commercial or financial relationships that could be construed as a potential conflict of interest.
