# Supplementary material for: Development of a Comorbidity-Based Nomogram to Predict Survival After Salvage Reirradiation of Locally Recurrent Nasopharyngeal Carcinoma in the Intensity-Modulated Radiotherapy Era
Source: Front Oncol. 2021 Jan 20;10:625184. doi: 10.3389/fonc.2020.625184 (PMC7855849; doi:10.3389/fonc.2020.625184)
Supplement: Supplementary file 1 [file Table_1.docx]

**Supplemental Table S1: the 5-year OS rate in locally recurrent nasopharyngeal carcinoma patients with rT3-4**

| chemotherapy regimens | 5-year OS rate (%) | | |
| --- | --- | --- | --- |
|  | rT3-4 | rT3-4(ACE-27 < 2) | rT3-4(ACE-27≥ 2) |
| RT | 16.7 | 17.1 | 15.0 |
| IC + RT | 20.7 | 22.5 | 14.0 |
| CCRT | 30.4 | 35.2 | 7.2 |
| IC + CCRT | 38.6 | 38.8 | 37.5 |

Abbreviations: RT, radiotherapy; IC, induction chemotherapy; CCRT; concomitant chemoradiotherapy; OS, overall survival.; ACE-27, Adult Comorbidity Evaluation-27.
