# Supplementary material for: Development of a Comorbidity-Based Nomogram to Predict Survival After Salvage Reirradiation of Locally Recurrent Nasopharyngeal Carcinoma in the Intensity-Modulated Radiotherapy Era
Source: Front Oncol. 2021 Jan 20;10:625184. doi: 10.3389/fonc.2020.625184 (PMC7855849; doi:10.3389/fonc.2020.625184)
Supplement: Supplementary file 2 [file Table_2.docx]

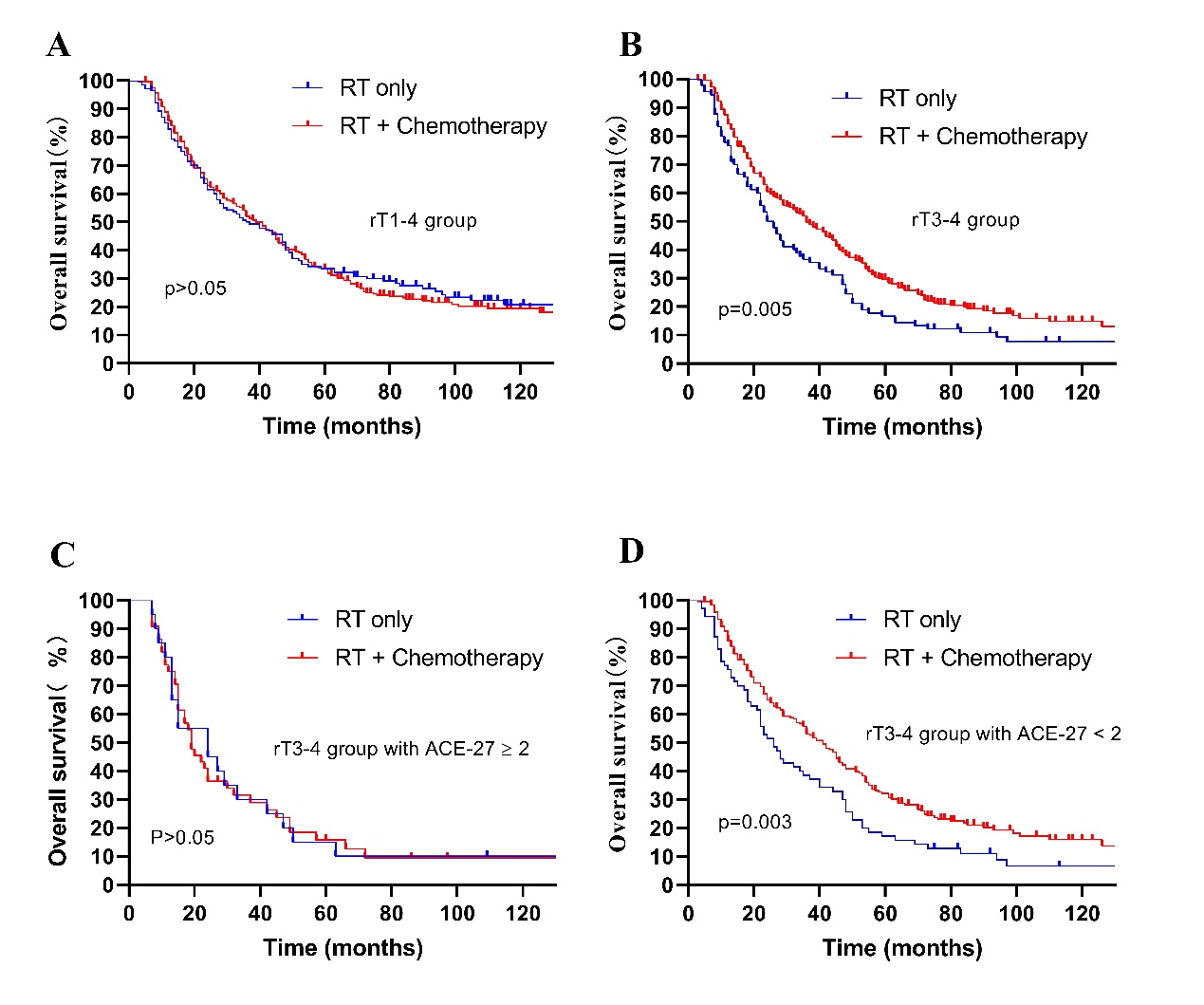


Supplemental Figure S1. Kaplan-Meier overall survival curves for patients with locally recurrent nasopharyngeal cancer. A: Overall survival for patients with radiotherapy (RT) only or chemotherapy plus radiotherapy; B: Overall survival for patients with recurrent T3-4 stage treated with radiotherapy (RT) only or chemotherapy plus radiotherapy; C: Overall survival for patients with recurrent T3-4 stage and ACE-27 ≥ 2 treated with radiotherapy (RT) only or chemotherapy plus radiotherapy; D: Overall survival for patients with recurrent T3-4 stage and ACE-27 < 2 treated with radiotherapy (RT) only or chemotherapy plus radiotherapy. ACE-27, Adult Comorbidity Evaluation 27; RT, radiotherapy.


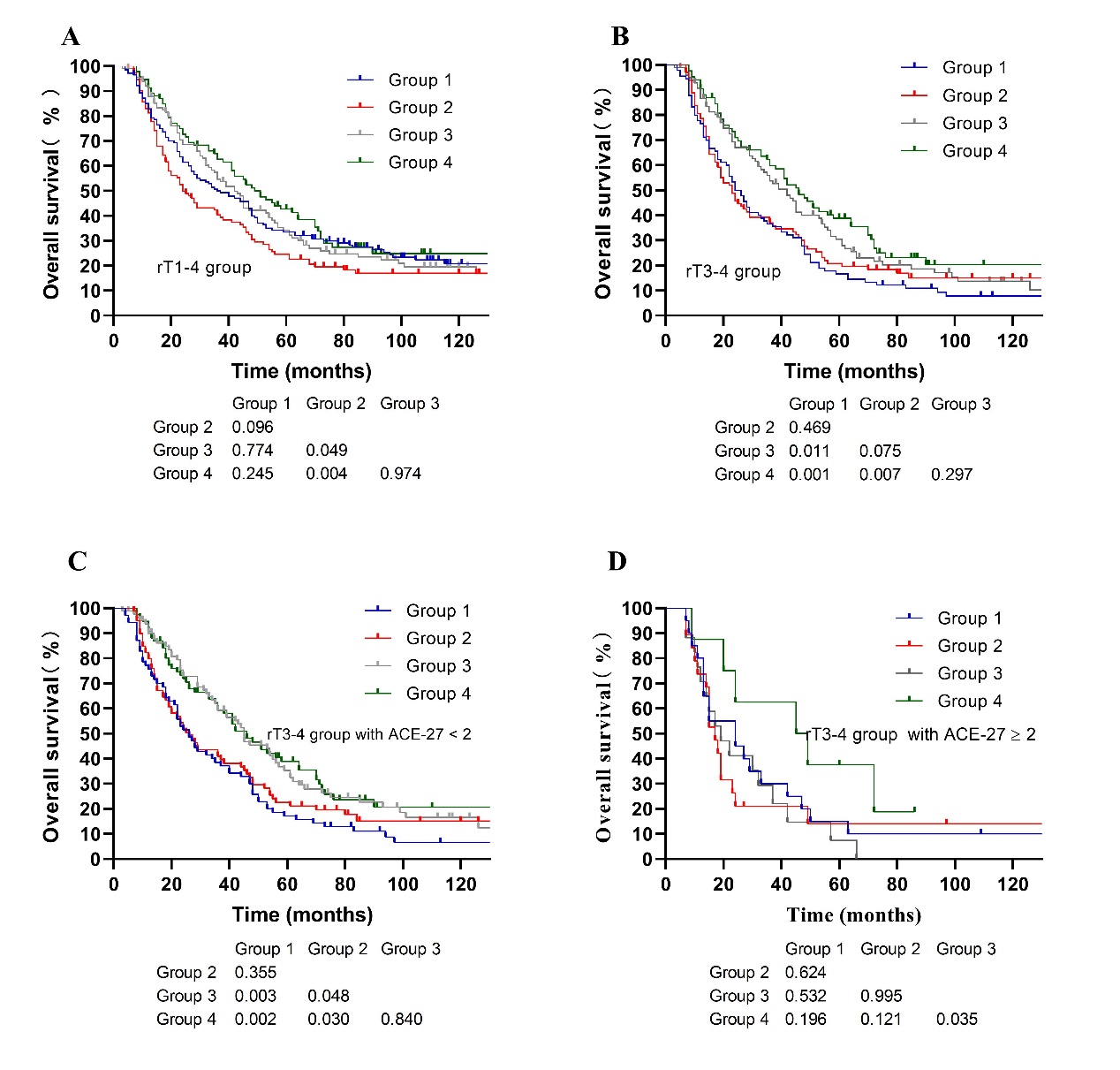


Supplemental Figure S2. Kaplan-Meier overall survival curves for patients with locally recurrent nasopharyngeal cancer according to the treatment strategy. A. Overall survival for patients treated with different strategy ; B, Overall survival for patients with recurrent T3-4 stage treated with different strategy; C, Overall survival for patients with recurrent T3-4 stage and ACE-27 < 2 treated with different strategy; D, Overall survival for patients with recurrent T3-4 stage and ACE-27 ≥ 2 treated with different strategy. Group 1: radiotherapy (RT) only; Group 2: radiotherapy plus induction chemotherapy (RT + IC): Group 3: concomitant chemoradiotherapy (CCRT); Group 4: concomitant chemoradiotherapy plus induction chemotherapy (CCRT + IC).


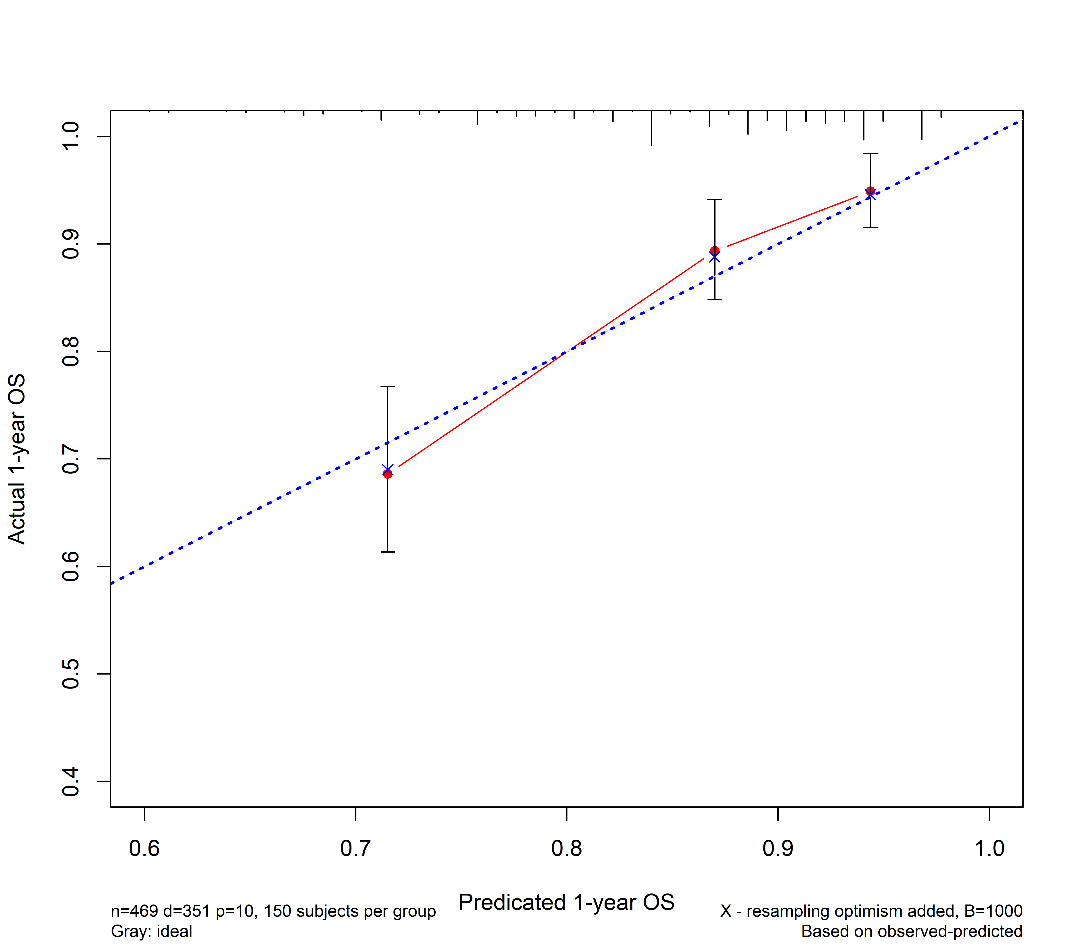


Supplemental Figure S3. The calibration curve of the nomogram for predicting the 1-year overall survival. The actual value is plotted on the Y-axis, and the nomogram-predicted probability of overall survival is plotted on the X-axis.


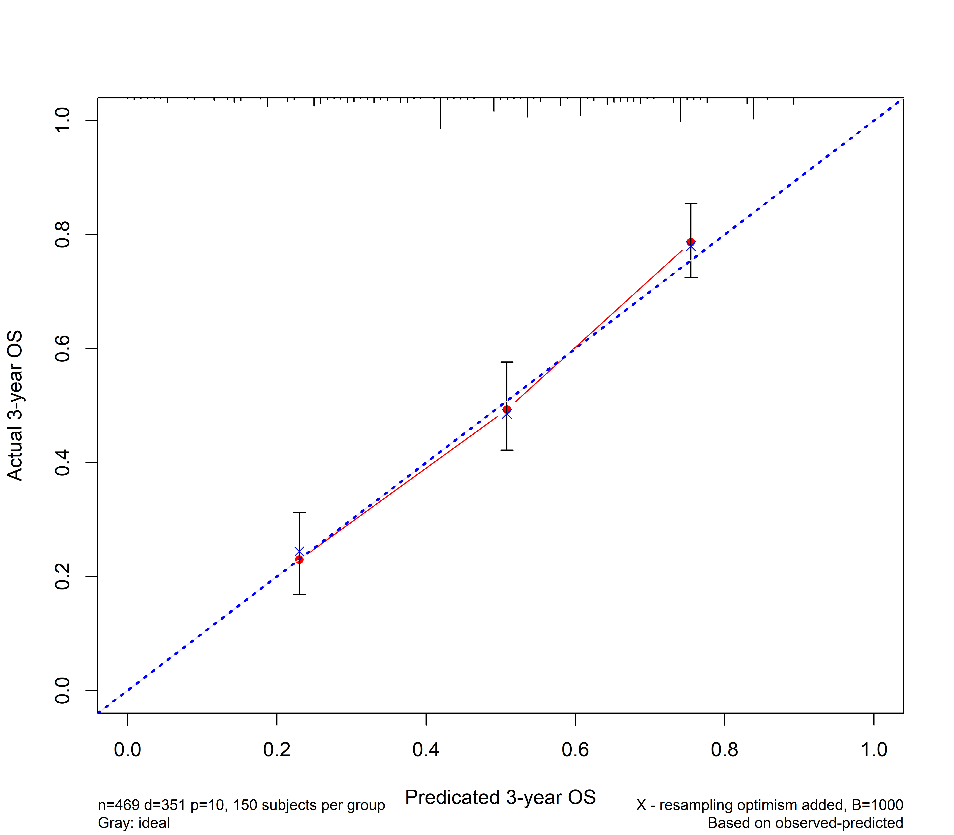


Supplemental Figure S4. The calibration curve of the nomogram for predicting the 3-year overall survival. The actual value is plotted on the Y-axis, and the nomogram-predicted probability of overall survival is plotted on the X-axis.


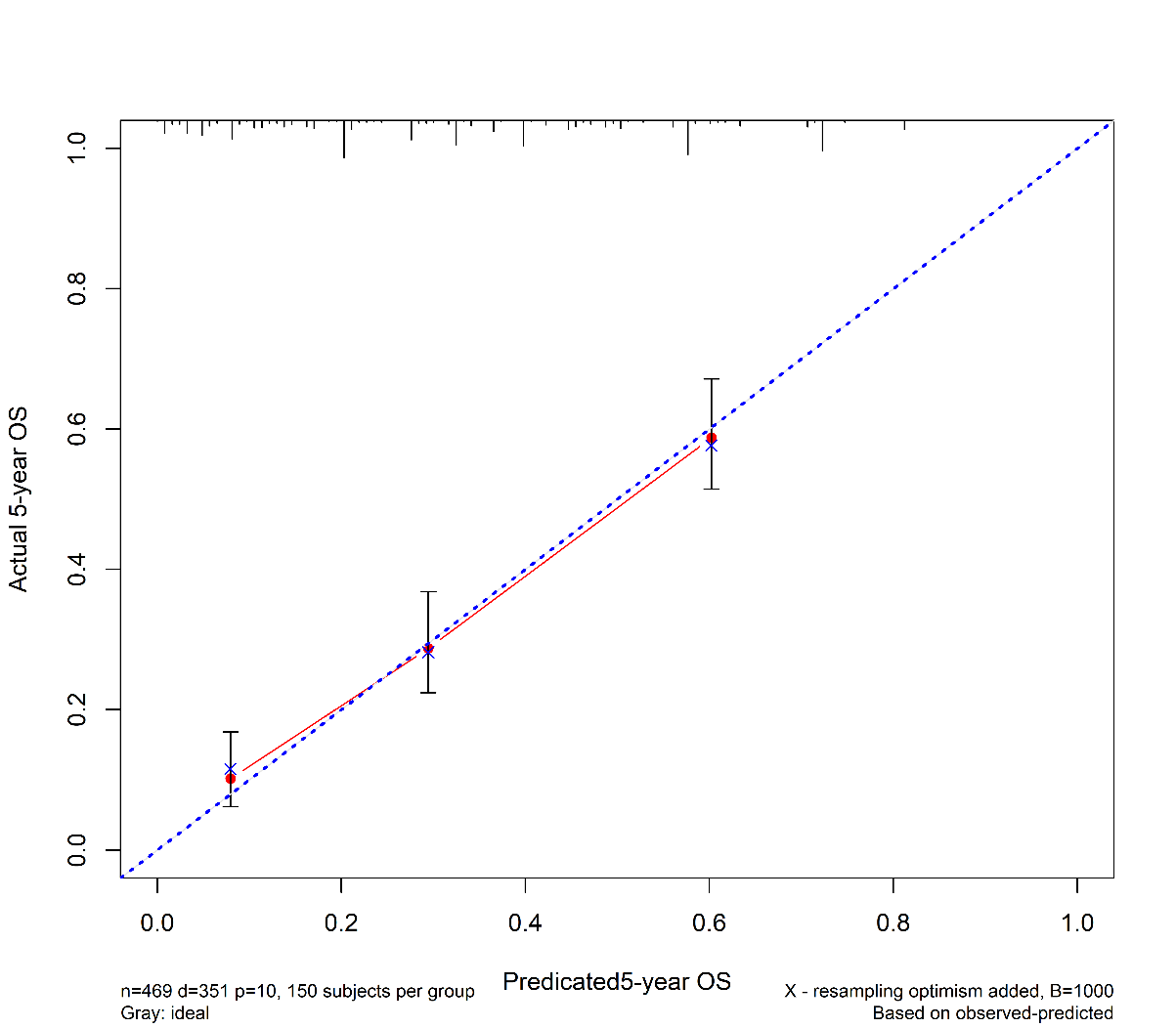


Supplemental Figure S5. The calibration curve of the nomogram for predicting the 5-year overall survival. The actual value is plotted on the Y-axis, and the nomogram-predicted probability of overall survival is plotted on the X-axis.
